
JOURNAL INFORMATION
==============================
NLM Title Abbreviation: ScientificWorldJournal
Journal ID: ScientificWorldJournal
NLM Journal ID: 101131163
Journal ID: TSWJ

The Scientific World Journal

ISSN: 2356-6140
EISSN: 1537-744X
Publisher: Wiley

ARTICLE INFORMATION
==============================
PMCID: PMC6009257
PMID: 29973853
DOI: 10.1100/tsw.2002.172
Article ID: 136531
Article version: 1
Subjects: Editorial

Isotopes as Tracers in Marine Environmental Studies – A Forthcoming Collection of Papers in the Isotopes in the Environment Domain

Povinec Pavel *
IAEA – Marine Environmental Laboratory, Monaco, France
*Pavel Povinec: p.povinec@iaea.org
Publication date: 2002
Electronic publication date: 2002 Apr 15
Volume: 2
First page: 1022
Last page: 1022
Copyright: Copyright © 2002 Pavel Povinec.
Copyright year: 2002
License: This is an open access article distributed under the Creative Commons Attribution License, which permits unrestricted use, distribution, and reproduction in any medium, provided the original work is properly cited.
License URL: https://creativecommons.org/licenses/by/3.0/

Keywords: isotopes, seawater, sediment, dating methods, modelling, environmental change, radiometrics, accelerator mass spectrometry, database

==============================
**Editorial**
Isotopes as Tracers in Marine Environmental Studies: Collected Papers from 2001: An Ocean Odyssey
*TheScientificWorldJOURNAL* (2002) 2, 1022
ISSN 1537-744X; DOI 10.1100/tsw.2002.172

TheScientificWorld
www.thescientificworld.com

### Isotopes as Tracers in Marine Environmental Studies – A Forthcoming Collection of Papers in the *Isotopes in the Environment* Domain

Pavel Povinec
*IAEA – Marine Environmental Laboratory, Monaco*

*Received February 15, 2002; Accepted February 15, 2002; Published April 15, 2002*

---

**KEY WORDS:** isotopes, seawater, sediment, dating methods, modelling, environmental change, radiometrics, accelerator mass spectrometry, database

**DOMAINS:** isotopes in the environment, marine systems, environmental chemistry

---

*TheScientificWorldJOURNAL* and the Isotopes in the Environment Domain announce the forthcoming publication of peer-reviewed papers on Isotopes as Tracers in Marine Environmental Studies, a session held at the '2001: An Ocean Odyssey Conference' on 21–28 October 2001, Mar Del Plata, Argentina.

The conference provided an opportunity to explore both radioactive and stable isotopes as tracers in studies of oceanographic processes, behaviour of radionuclides in the water column, water and sediment dynamics, marine environmental change, and computer modelling of dispersion of isotopes in the marine environment.

The Guest Editor for the collection is Pavel Povinec and we expect publication of around 15 papers to be completed by March 2002. Each paper will be promoted to all relevant Domains of *TheScientificWorldJOURNAL* – an interdisciplinary approach much in line with the general objectives of the conference. Readers are invited to check back regularly to the Table of Contents for the Collection on *TheScientificWorldJOURNAL* website, as papers are published continuously upon review and acceptance.

---

**This article should be referenced as follows:**

Povinec, P. (2002) Isotopes as tracers in marine environmental studies — a forthcoming collection of papers in the *Isotopes in the Environment* domain. In Isotopes as Tracers in Marine Environmental Studies: Collected Papers from 2001: An Ocean Odyssey. *TheScientificWorldJOURNAL* **2**, 1022.

---

Email: P.Povinec@iaea.org
©2002 with author.
